# New surgical technique of laparoscopic resection of adenomyosis under real-time intraoperative ultrasound elastography guidance: A case report

**DOI:** 10.1016/j.heliyon.2020.e04628

**Published:** 2020-08-04

**Authors:** Yoshiaki Ota, Kuniaki Ota, Toshifumi Takahashi, Soichiro Suzuki, Rikiya Sano, Mitsuru Shiota

**Affiliations:** a Department of Gynecologic Oncology, Kawasaki Medical School, Okayama, 701-0192, Japan; b Fukushima Medical Center for Children and Women, Fukushima Medical University, Fukushima, 960-1295, Japan

**Keywords:** Abdominal surgery, Gynecology, Medical imaging, Pregnancy, Reproductive system, Adenomyosis, Laparoscopic surgery, Ultrasonography, Elastography, Fertility preservation

## Abstract

Detecting adenomyosis in the myometrium is a challenge since it is infiltrative with ill-defined margins and can be often confused with uterine fibroids. However, recent advances, such as ultrasound elastography, have enabled its detection in the myometrium, thereby facilitating its accurate diagnosis. We report our experience of performing complete laparoscopic resection of adenomyosis under real-time ultrasound elastography guidance in a 32-year-old woman who underwent laparoscopic adenomyomectomy following severe dysmenorrhea and heavy menstrual bleeding. Real-time ultrasound elastography was also utilized intraoperatively to detect residual adenomyosis. Complete adenomyosis resection and uterine reconstruction were achieved. Follow-up magnetic resonance imaging was conducted to confirm successful uterine reconstruction. The patient recovered rapidly with no complications. Intraoperative elastography-guided laparoscopic adenomyomectomy was feasible and effective in completely removing adenomyotic lesions.

## 1 Introduction

Typically, adenomyosis affects the endo-myometrial junction, which has heterotopic endometrial glands and myometrial stroma with ill-demarcated margins [1]. Even advanced imaging techniques such as high-resolution ultrasound and magnetic resonance imaging (MRI) have proven to be ineffective for detecting adenomyosis, especially in identifying its extent and localization [2, 3]. Uncertainty in defining the site and extent of adenomyosis makes it difficult to determine the feasibility and accuracy of complete excision when conserving the uterus [4]. This is one reason why hysterectomy has remained the most common surgical intervention for treating adenomyosis.

Elastography is increasingly being used for the diagnosis of various diseases. It is an objective quantitative ultrasound technique, which can depict the stiffness of anatomical structures, such as myometrial tissue, to aid in their detection and characterization [5]. It was recently applied in several studies to diagnose adenomyosis and uterine leiomyomas [6, 7]. However, to our knowledge, no attempts at using real-time ultrasound elastography guidance for detecting residual adenomyosis and performing complete laparoscopic adenomyomectomy have been reported. We report the first case in which real-time ultrasound elastography was applied during complete laparoscopic adenomyomectomy.

## 2 Case presentation

**Figure 1:**
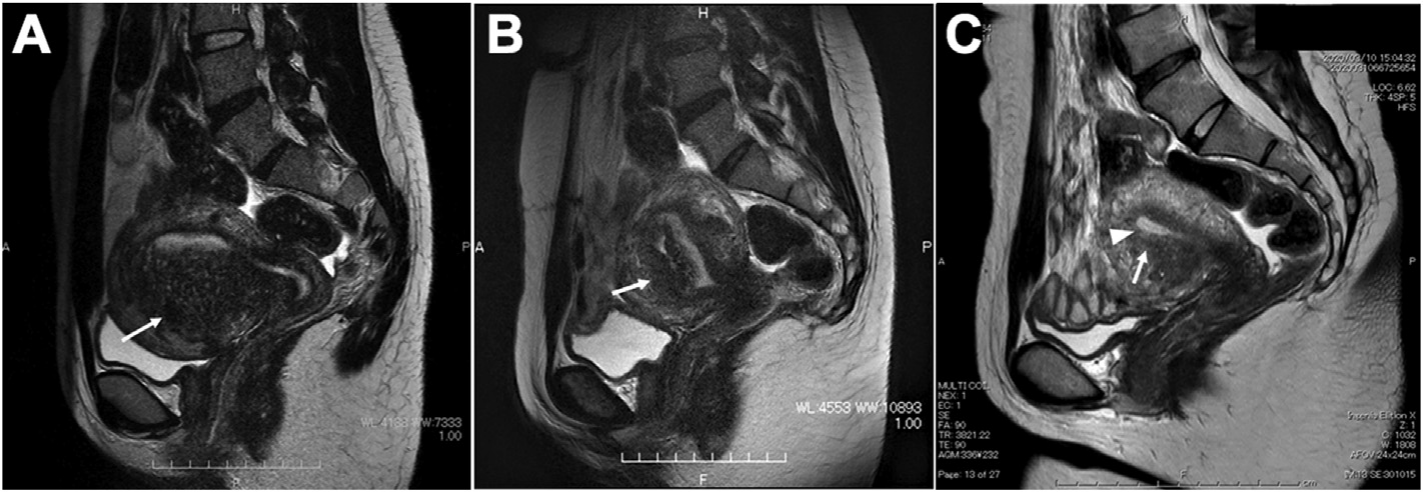
Magnetic resonance imaging (MRI) of pre- and post-laparoscopic adenomyomectomy. A: Sagittal T2-weighted preoperative pelvic MRI. Image shows substantial adenomyosis in the anterior myometrium with a remarkably thickened junctional zone (white arrows). B: Sagittal T2-weighted postoperative pelvic MRI (4 weeks later). Image shows no residual adenomyosis. Note the complete uterine wound-healing (white arrow). C: Sagittal T2-weighted postoperative pelvic MRI (12 weeks later). Image shows normal thickness of the junctional zone (white arrow) and a regular endometrial cavity with no defect visible (arrowhead).

The patient provided written consent for publication. A 32-year-old nulliparous woman had experienced severe dysmenorrhea and heavy menstrual bleeding, which were treated with long-term dienogest for 2 years. Subsequently, her medication was discontinued after she decided to conceive. However, she could not achieve successful conception and underwent in vitro fertilization (IVF) treatment. Thereafter, preoperative embryo-cryopreservation and endoscopic surgery were selected for the patient in order to manage the risks of implantation failure due to a recurrence of adenomyosis. She underwent IVF followed by embryo-cryopreservation; laparoscopic surgery was scheduled. The patient has received a dienogest during the waiting period after IVF treatment until laparoscopic surgery. Preoperative two-dimensional transvaginal ultrasonography showed a thickened myometrium sized 6 × 5 cm in the anterior wall of the uterus. The thickening of the muscle layer was diffuse and heterogenous echogenic findings with poor definition of the junctional zone. Myometrial cyst was not observed in the thickened myometrium by two-dimensional transvaginal ultrasonography. The preoperative T2-weighted MRI image showed diffuse thickening of the junctional zone in the anterior wall (Figure 1A). The length of the thickening of the junctional zone was 5 cm, which was corresponded to the ratio of the junctional zone maximum/total myometrium was 77%.

**Figure 2:**
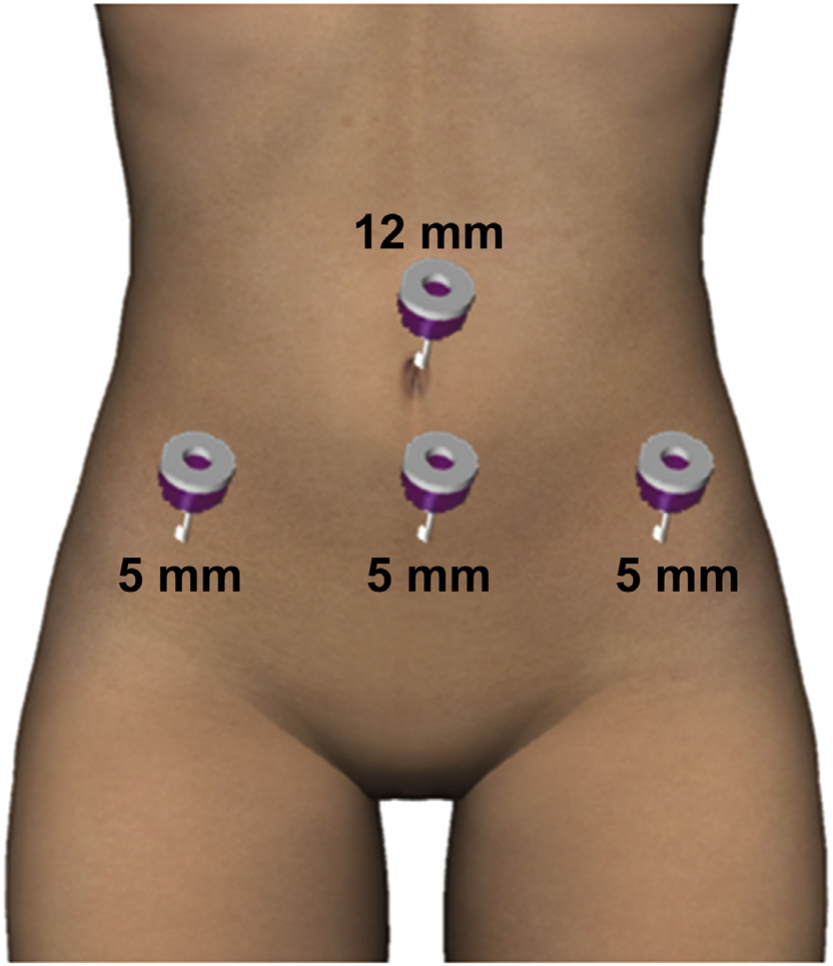
Placement of trocar ports on the patient's abdomen. A 12-mm port for the zero-degree laparoscope was introduced intra-umbilically; three additional 5-mm lateral ports were placed under direct vision, centrally and on the left and right lateral sides.

**Figure 3:**
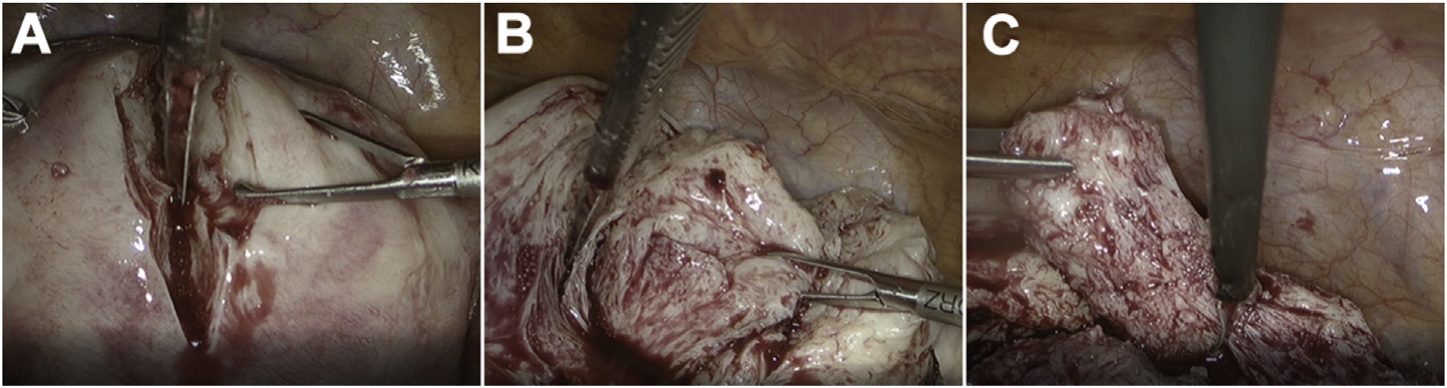
Laparoscopic adenomyomectomy procedure. (A) The uterus is longitudinally incised with the scalpel to access the adenomyotic tissue. (B) The nucleation is resected in a wide wedge-shape in the anterior uterine wall and fundus. (C) The adenomyotic tissues close to the endometrium are excised with scissors forceps.

**Figure 4:**
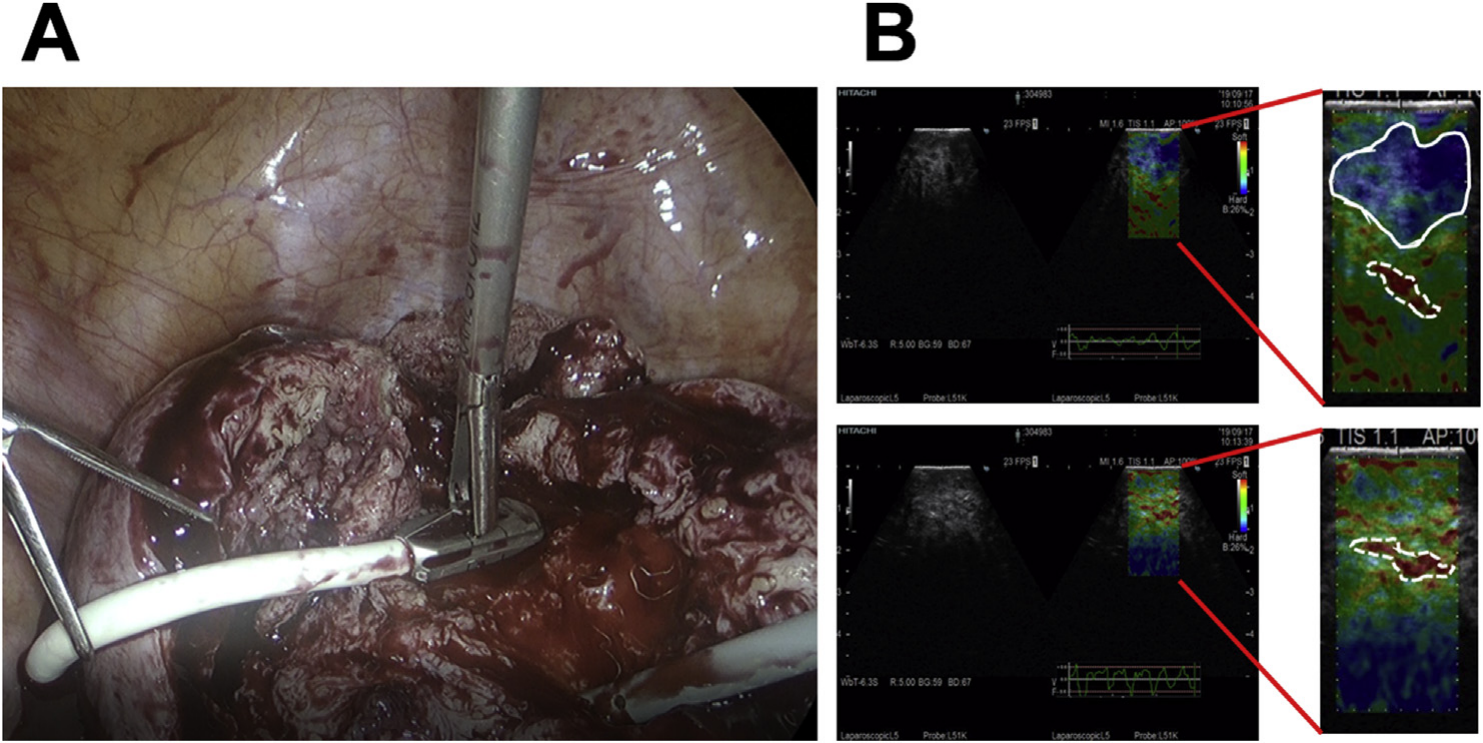
A: A laparoscopic probe is applied via the dissected uterine wall to detect any residual adenomyosis. B: Intraoperative real-time tissue elastography shows residual adenomyosis, indicated by the bright blue areas (white solid circle), and the endometrium, indicated by the bright red areas (white dotted circle) (top). After complete resection, no bright blue areas are detected by real-time tissue elastography (bottom).

Laparoscopic surgery was performed under general anesthesia in the Trendelenburg position with a four-port, 2D/4K laparoscopy system (VISERA 4K UHD Camera Control Unit; Olympus Medical System, Tokyo, Japan). A 12-mm port (ENDOPATH XCEL®, Ethicon Endo-Surgery, Cincinnati, USA) for the zero-degree laparoscope was introduced intra-umbilically; three additional 5-mm lateral ports (ENDOPATH XCEL®, E Ethicon Endo-Surgery, Tokyo, Japan) were placed under direct vision, centrally and on the left and right lateral sides (Figure 2). The surgeon used the central and left-sided lateral ports to perform most of the surgical procedures. Laparoscopic findings showed a fist-sized enlarged uterus and the uterus and bilateral adnexa showed no pelvic adhesions. Tubal patency was not examined because of scheduled frozen-thawed embryo transfer. Vasopressin (diluted at 1 IU/70 ml normal saline) was injected into the uterine wall to decrease intraoperative bleeding. A number 11 surgical scalpel (Feather Safety Razor Co. Ltd., Tokyo, Japan) was inserted directly into the abdominal cavity after removing the trocar placed between the pubis symphysis and the umbilicus; a longitudinal incision was performed over the uterine fundus (Figure 3A). Adenomyotic lesions were carefully excised with the surgical scalpel along the anterior wall of the uterus (Figure 3B, C). If the myometrium appeared normal, it was spared maximally. Areas of persistent bleeding were managed using a bipolar electrosurgical device. After initial enucleation of the adenomyosis, we decided to use a laparoscopic ultrasound probe to detect the remaining adenomyosis part. Because a laparoscopic ultrasound probe can only be inserted through a 12 mm trocar, the cul-de-sac was selected as the site of insertion. A posterior 1–2-cm transverse colpotomy was performed laparoscopically, precisely in the midline of the posterior fornix, demarcated using a Vagi-Pipe® (Hakko Medical, Nagano, Japan). The laparoscopic ultrasound probe (ARIETTA 850, Hitachi, Ltd., Tokyo, Japan) was inserted from the colpotomy incision (Figure 4A); intraoperative real-time tissue elastography revealed residual adenomyosis in a small part of the uterine wall, depicted as bright blue areas (Figure 4B). The residual adenomyotic tissues were completely resected through frequent reassessment by real-time elastography. After enucleating the adenomyotic tissues maximally, the resected specimen was removed through the colpotomy incision into the vagina. The endometrium was ruptured and the uterine cavity was exposed during excision of the adenomyotic lesions. The endometrium was sutured with 3–0 absorbable monofilament polydioxanone (3-0 PDS® II, Ethicon Endo-Surgery, Tokyo, Japan). The uterine incisions were repaired in multiple layers using barbed sutures (0 Stratafix® Symmetric PDS® plus, Ethicon Endo-Surgery, Tokyo, Japan). After the myometrium was reapproximated and hemostasis was achieved, the serosal layer was closed using a synthetic absorbable suture (1-0 Vicryl®, Ethicon Endo-Surgery, Tokyo, Japan) on a CT-1 needle. There were no intraoperative complications. The operation lasted 127 min, and the estimated blood loss was 450 mL (Supplemental video). The patient had an uneventful immediate postoperative recovery period. Four-week and 12-week postoperative MRI revealed complete uterine reconstruction (Figure 1B, C).

Supplementary content related to this article has been published online at https://doi.org/10.1016/j.heliyon.2020.e04628.

The following are the supplementary data related to this article:

Video. 1**A movie of intraoperative elastography-guided laparoscopic adenomyomectomy.** A 32-year-old infertility woman underwent laparoscopic adenomyomectomy due to severe dysmenorrhea and heavy menstrual bleeding. Real-time ultrasound elastography was utilized intraoperatively to detect residual adenomyosis. Complete adenomyosis resection and uterine reconstruction were achieved.

## 3 Discussion

We performed a complete laparoscopic adenomyomectomy with intraoperative ultrasound elastography. To the best of our knowledge, this is the first report on the application of elastography for the detection of residual adenomyosis and its complete enucleation.

Essentially, ultrasonography and MRI are used for the diagnosis of adenomyosis; transvaginal ultrasonography (TVUS) is a first-line test, while MRI is a second-line imaging modality used for localization of adenomyosis. A recent review described that the pooled sensitivity and specificity in the diagnosis of adenomyosis were 0.72 and 0.82 for TVUS and 0.77 and 0.89 for MRI, respectively [2]. In cases when the uterus is enlarged or fibroids coexist, MRI was proven to have a higher sensitivity than TVUS in discriminating between adenomyosis and fibroids [8]. However, MRI cannot detect the diffuse form, which involves the dissemination of the adenomyotic foci throughout the uterus [2].

Elastography is an emerging imaging modality used for detection of adenomyosis; it quantifies the local mechanical properties of tissues and evaluates their stiffness. There are differences in the tissue stiffness of the normal myometrium, a fibroid, and adenomyosis. Therefore, this imaging modality was used to diagnose adenomyosis by detecting the characteristic tissue stiffness in recent reports. Zhang et al. reported the possibility of detecting adenomyosis with elastography based on the globally increased myometrial stiffness caused by the disease [6]. Stoelinga et al. stated that the confirmed diagnosis of adenomyosis by ultrasound elastography correlated with histology and MRI [7]. However, the detection of residual adenomyotic lesions during adenomyomectomy has not yet been reported.

Adenomyomectomy involves several surgical procedures, such as resection and wound closure. The surgical techniques are complex, and there is no agreement on the optimal incision, closure, and suturing methods. Thus, surgeons tend to perform open adenomyomectomy rather than laparoscopic surgery. This is because during laparoscopic surgeries, only limited directions of movement and instrumentation use can be accommodated; in addition, palpation is impossible. Diffuse-type lesions, however, require extensive resection and complicated suturing, necessitating difficult procedures involving advanced techniques [9]. Furthermore, the laparoscopic approach has higher risks of uterine rupture due to overreaction by short, weak contractions, which are not faced during laparotomy [10]. However, since laparoscopic surgery is minimally invasive, laparoscopic adenomyomectomy may be acceptable.

Complete removal of adenomyotic lesions is technically challenging because of the ill-defined margins between the adenomyotic lesions and normal myometrial tissue. In particular, for a case of laparoscopic myomectomy, intraoperative ultrasonography was used to identify residual fibroids in a previous report [11]. However, ultrasonography has not been used in the surgical treatment of adenomyosis. Therefore, we utilized real-time ultrasound elastography to detect any residual adenomyosis during laparoscopic surgery. We performed a conventional ultrasound scan followed by ultrasound elastography during adenomyomectomy (Figure 4B). In the conventional scan, it was difficult to distinguish between normal myometrium and residual lesions of adenomyosis, but elastography was able to distinguish residual adenomyotic lesion with color mapping. Color mapping of the lesions was very useful for removing residual adenomyotic tissues. In addition, the ultrasound probe for elastography inserted through the cul-de-sac is designed to be easily grasped with laparoscopic forceps, allowing sufficient pressure to be applied to the tissue for stiffness measurement. Thus, real-time intraoperative ultrasound elastography could potentially identify the lesion's margins, allowing complete resection with minimal damage to the surrounding healthy tissue.

There is no clear consensus for permitting pregnancy after adenomyomectomy, as with myomectomy, the extent of myometrial repair should be evaluated. At present, there is no reliable index for evaluating repair of the muscle layer, but it is considered that one index is the restoration of blood flow without hematoma formation in the enucleated site by postoperative ultrasonography and MRI examination [12].

Although uterine-conserving operative treatment of adenomyosis is feasible and can be efficacious, uterine rupture during pregnancy remains a serious concern after adenomyomectomy. Thus, this technique must be used in carefully selected patients with the correct indications. Further guidelines and long-term follow-up after using real-time ultrasound elastography will help determine the role of this promising new technique in avoiding residual adenomyosis and improving fertility-conserving surgery.

## Declarations

### Author contribution statement

All authors listed have significantly contributed to the investigation, development and writing of this article.

### Funding statement

This research did not receive any specific grant from funding agencies in the public, commercial, or not-for-profit sectors.

### Competing interest statement

The authors declare no conflict of interest.

### Additional information

No additional information is available for this paper.
