# Corrigendum to “New surgical technique of laparoscopic resection of adenomyosis under real-time intraoperative ultrasound elastography guidance: A case report”[Heliyon 6 (8), (August 2020) Article 04628]

**DOI:** 10.1016/j.heliyon.2020.e04868

**Published:** 2020-09-07

**Authors:** Yoshiaki Ota, Kuniaki Ota, Toshifumi Takahashi, Soichiro Suzuki, Rikiya Sano, Mitsuru Shiota

**Affiliations:** a Department of Gynecologic Oncology, Kawasaki Medical School, Okayama, 701-0192, Japan; b Fukushima Medical Center for Children and Women, Fukushima Medical University, Fukushima, 960-1295, Japan

In the original published version of this article, the supplementary video showed the patient's identification number. This has now been removed. The authors apologize for this error. Both the HTML and PDF versions of the article have been updated to correct the error.

